# Assessment of carbapenem-resistant *Acinetobacter baumannii*–colonized patients: Which specimens produce the highest yield?

**DOI:** 10.1017/ash.2023.370

**Published:** 2023-09-29

**Authors:** Casey Morrell, Kristina McClanahan, Lauren Daniel, James Burks, Argentina Charles, Ashley Marin, Jeanne Negley, Melanie Roderick, Carolyn Stover

## Abstract

**Background:** Carbapenem-resistant *Acinetobacter* (CRA) bacteria are an urgent public health threat. Accurate and timely testing of CRA is important for proper infection control practices to minimize spread. In 2017, the CDC estimated 8,500 CRA cases among hospitalized patients, 700 deaths, and $281 million in attributable healthcare costs. Treatment options are extremely limited for carbapenem-resistant *Acinetobacter baumannii* (CRAB) infections, making CRAB a unique concern. Colonization screening is a valuable tool for containment but requires sampling of 4 body sites. Identifying a reliable specimen collection site for CRAB is important to inform public health recommendations as screening can cost healthcare facilities valuable time and resources. **Methods:** Results of all screening specimens of patients with at least 1 site positive for CRAB on a unique collection date were extracted from the Southeast Regional data of Antimicrobial Resistance Lab Network (SEARLN) data. Non-CRAB screening and screenings that did not yield at least 1 positive result on a single collection date were excluded. We also limited our data to include only the following screening sites, which have been validated by the Tennessee Department of Health’s State Public Health Laboratory: axilla and groin, rectal, sputum, and wound. For each specimen source, we calculated the percentage of positive specimen among CRAB-colonized patients. Data were extracted and analyzed using SAS version 9.4 software. **Results:** The SEARLN data contained 594 CRAB screening specimens collected over 4 years, 2018 through 2021, and 486 of those specimens yielded CRAB. For CRAB-colonized patients screened in this study, wound specimens had the highest positivity rate at 93.4% (95% CI, 89.9%–96.9%) of samples culturing CRAB. Sputum followed at 87.7%, then axilla and groin at 77.6% and rectal at 59.7%. **Conclusions:** Wound specimens produced the highest proportion of positive cultures among CRAB-positive patients, making them the sample type with the highest prevalence in our study. For healthcare facilities with limited time and resources seeking to optimize their CRAB screening process, wound specimens may be the most reliable single site for detecting CRAB colonization in patients with an open wound. When a wound is not present, sputum may be a good alternative single-source collection site. More research should be conducted before CRAB screening recommendations are updated.

**Disclosures:** None

